# Supplementary material for: Rapid determination of solid-state diffusion coefficients in Li-based batteries via intermittent current interruption method
Source: Nat Commun. 2023 Apr 21;14:2289. doi: 10.1038/s41467-023-37989-6 (PMC10121696; doi:10.1038/s41467-023-37989-6)
Supplement: Supplementary file 1 — Supplementary information [file 41467_2023_37989_MOESM1_ESM.pdf]

Supplementary Information

Rapid determination of solid-state diffusion coefficients in Li-based batteries via intermittent current interruption method

Yu-Chuan Chien[1,2], Haidong Liu[1], Ashok S. Menon[1,3], William R. Brant[1], Daniel Brandell[1,*], Matthew J. Lacey[4,*]

[1]Department of Chemistry—Ångström Laboratory, Uppsala University, Box 538, Lägerhyddsvägen 1, 751 21 Uppsala, Sweden

[4]Scania CV AB, 151 87 Södertälje, Sweden

[2]Present address: Breathe Battery Technologies, Office 7, 35-37 Ludgate Hill, London EC4M 7JN, United Kingdom

[3]Present address: WMG, University of Warwick, Coventry CV4 7AL, United Kingdom

*Correspondence should be addressed to D.B. (email: daniel.brandell@kemi.uu.se) or to M.J.L. (email: matthew.lacey@scania.com)

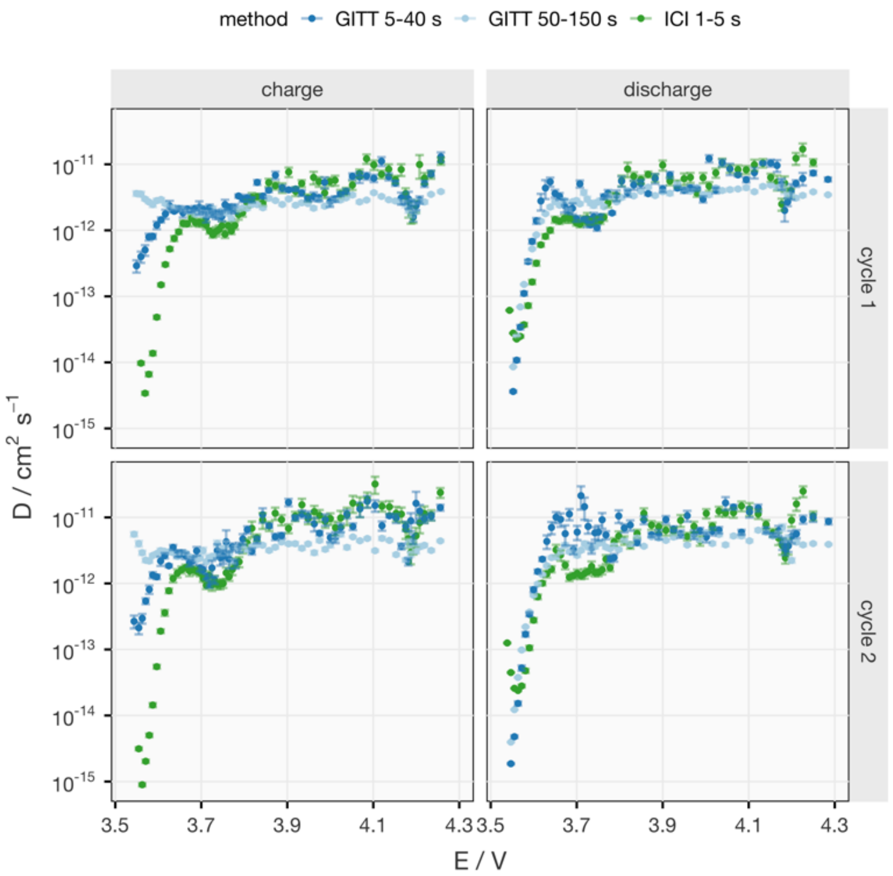

Supplementary Figure 1. Comparison between the Li-ion diffusion coefficient derived from the GITT, ICI method and EIS with the data of Cell 1.

The Li-ion diffusion coefficient in NMC811(D) in Cell 1 at various OCP of the electrode (E) against Li/Li^+ derived from the GITT with data selection interval 5–40 s and 50–150 s, the ICI method with data selection interval 1–5 s.

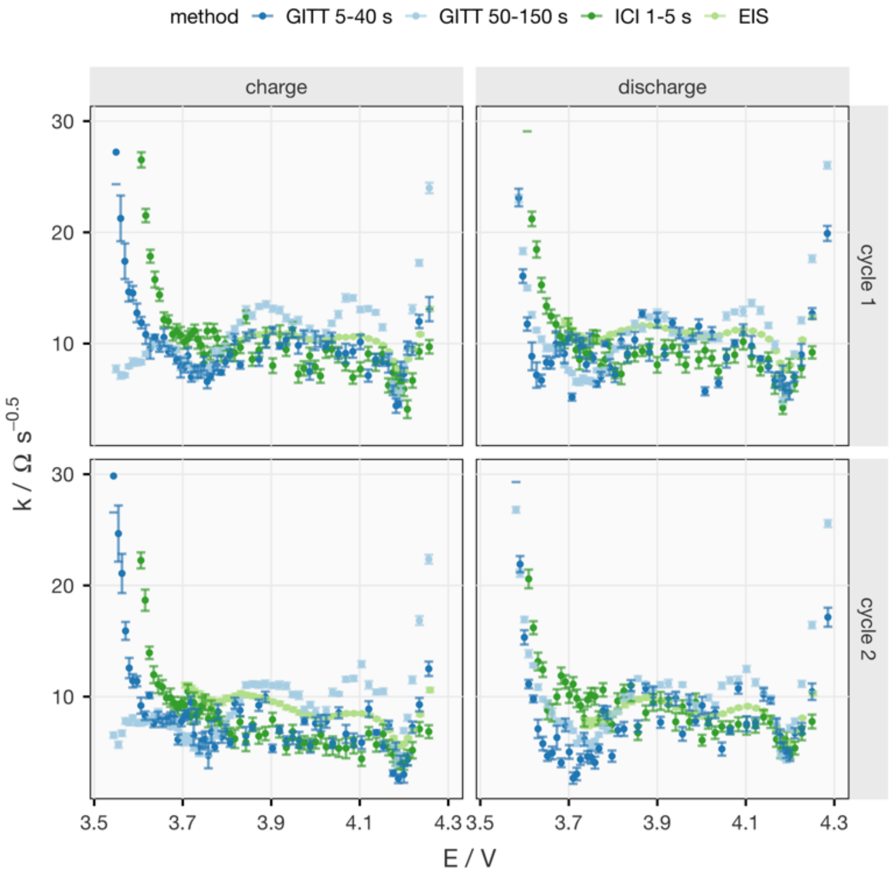

Supplementary Figure 2. Comparison between the diffusion resistance coefficient derived from the GITT, ICI method and EIS with the data of Cell 1.

The diffusion resistance coefficient (k) in NMC811(D) in Cell 1 at various OCP of the electrode (E) against Li/Li^+ derived from the GITT with data selection interval 5–40 s and 50–150 s, the ICI method with data selection interval 1–5 s and the EIS fitting ($k = \sigma\sqrt{8/\pi}$, σ: Warburg coefficient).

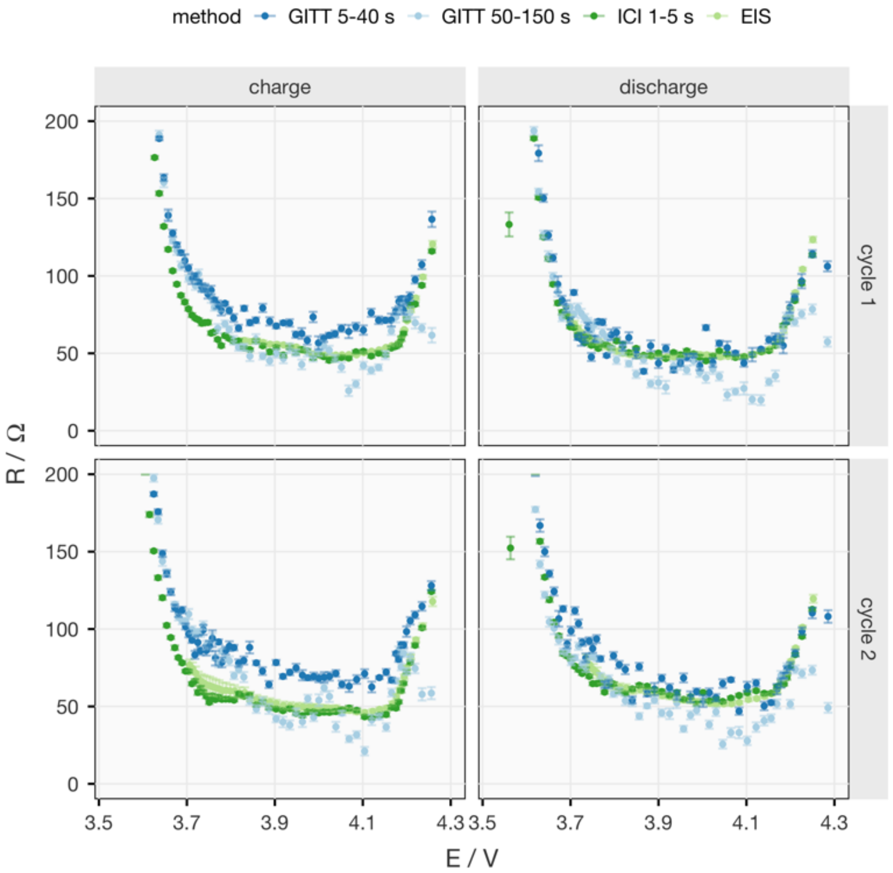

Supplementary Figure 3. Comparison between the internal resistance derived from the GITT, ICI method and EIS with the data of Cell 1.

The internal resistance (R) of NMC811in cell 1 at various OCP of the electrode (E) against Li/Li^{+} derived from the GITT with data selection interval 5–40 s and 50–150 s, the ICI method with data selection interval 1–5 s and the EIS fitting (R0+R1+R2 in Supplementary Fig. 13).

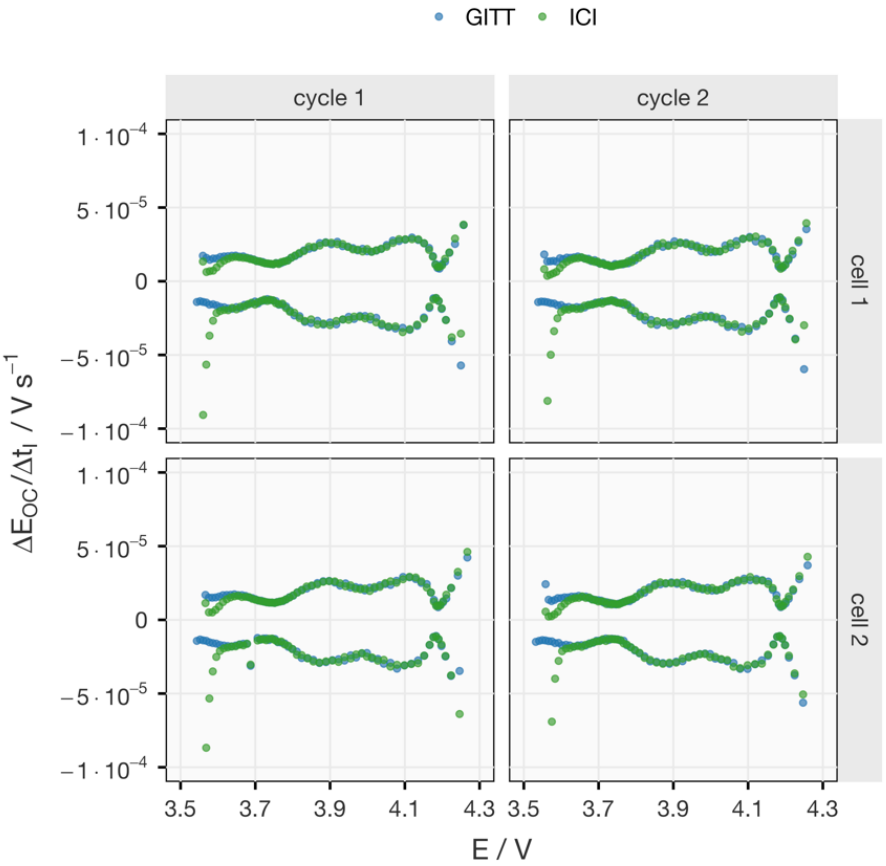

Supplementary Figure 4. Comparison between the slope of OCP derived from the GITT and ICI method with data from both Cell 1 and 2 in both cycles.

Two sources of the OCP slope ($\Delta E_{OC}/\Delta t_I$), relaxed potentials of the GITT (GITT) and iR-corrected pseudo-OCP provided by the ICI method (ICI), are plotted versus OCP of the electrode (E) against Li/Li^+.

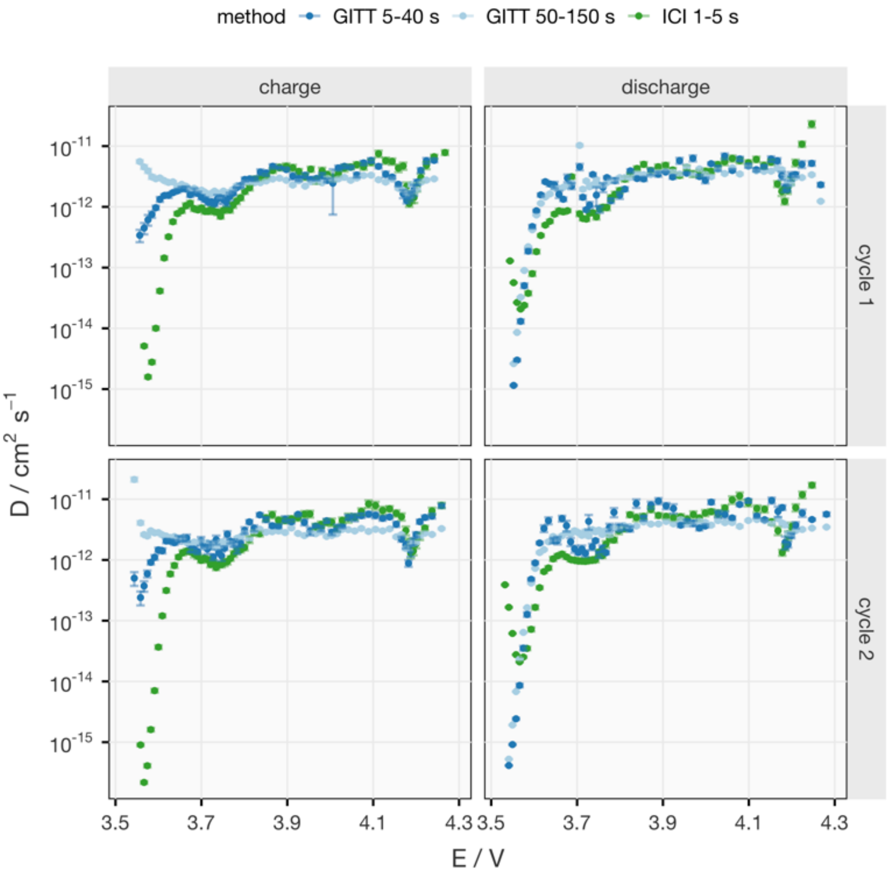

Supplementary Figure 5. Comparison between the Li-ion diffusion coefficient derived from the GITT, ICI method and EIS with the data of Cell 2.

The Li-ion diffusion coefficient in NMC811(D) in Cell 1 at various OCP of the electrode (E) against Li/Li^+ derived from the GITT with data selection interval 5–40 s and 50–150 s, the ICI method with data selection interval 1–5 s.

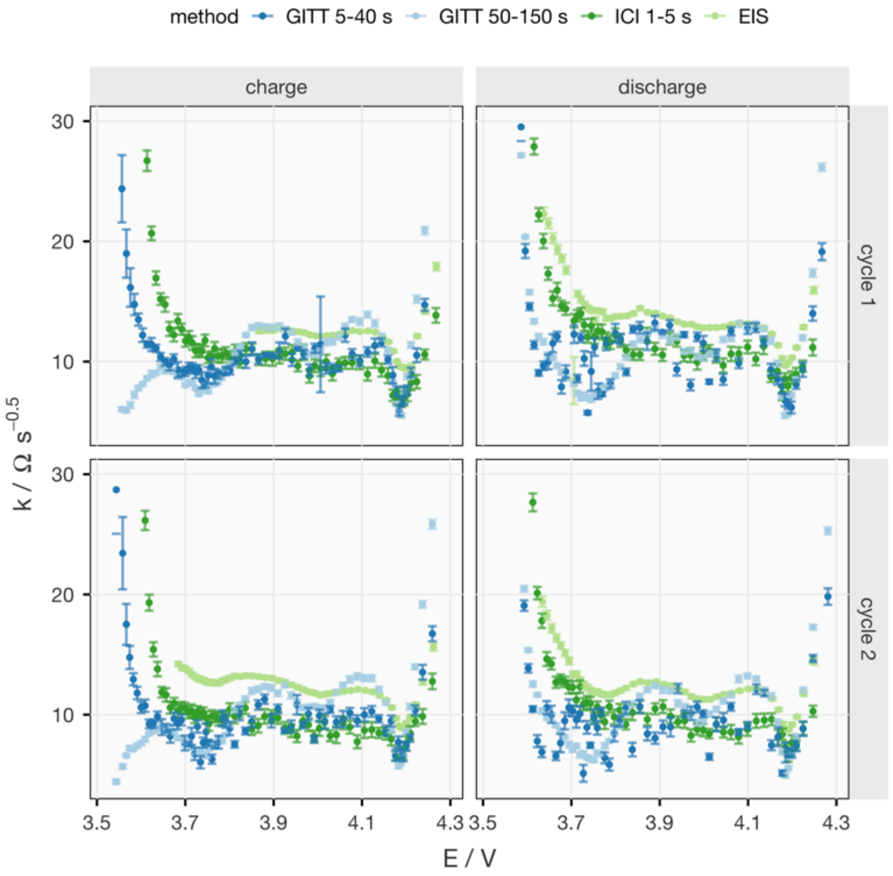

Supplementary Figure 6. Comparison between the diffusion resistance coefficient derived from the GITT, ICI method and EIS with the data of Cell 2.

The diffusion resistance coefficient (k) in NMC811(D) in Cell 1 at various OCP of the electrode (E) against Li/Li^+ derived from the GITT with data selection interval 5–40 s and 50–150 s, the ICI method with data selection interval 1–5 s and the EIS fitting ($k = \sigma\sqrt{8/\pi}$, σ: Warburg coefficient).

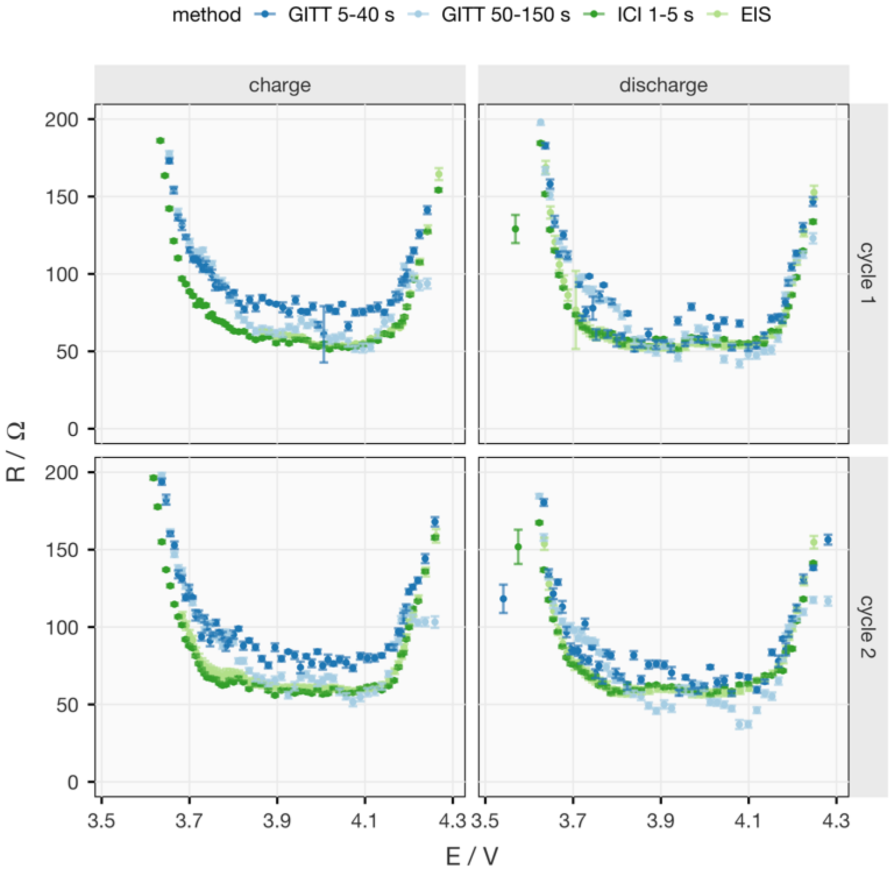

Supplementary Figure 7. Comparison between the internal resistance derived from the GITT, ICI method and EIS with the data of Cell 2.

The internal resistance (R) of NMC811in cell 1 at various OCP of the electrode (E) against Li/Li^{+} derived from the GITT with data selection interval 5–40 s and 50–150 s, the ICI method with data selection interval 1–5 s and the EIS fitting (R0+R1+R2 in Supplementary Fig. 13).

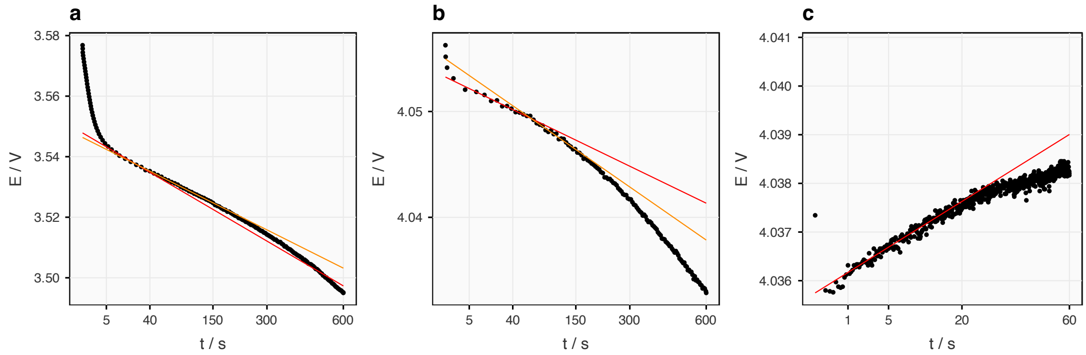

Supplementary Figure 8. Examples of GITT and ICI measurements

The electrode potential (E) is plotted against the step time (t) in the square root scale for all subplots. In the two examples of GITT measurements **a** below 3.7 V and **b** above 3.7 V, results from linear regression of potential against the square root of step time with datapoints in 5–40 s and 50–150 s intervals are also plotted as red and orange lines, respectively. **c** is an example of an ICI measurement, which took place after the GITT measurement in **b**. The red line shows the result from linear regression of potential against the square root of step time with datapoints in 1–5 s interval.

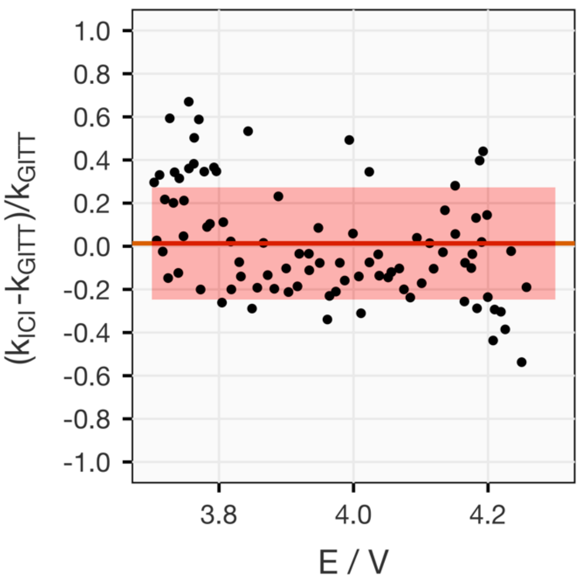

Supplementary Figure 9. Statistical analysis on the relative difference between the *k* values from the GITT and ICI methods.

The relative difference between the *k* values from the GITT and ICI methods (k_{GITT} and k_{ICI}, respectively) above 3.7 V in Figure 3 in the main text (Cell1 in cycle 1) plotted against the OCP of the electrode (E) against Li/Li^+. The average, 0.013, is shown by the horizontal line and the standard deviation, 0.26, is shown by the height of the shaded area above and below the average value.

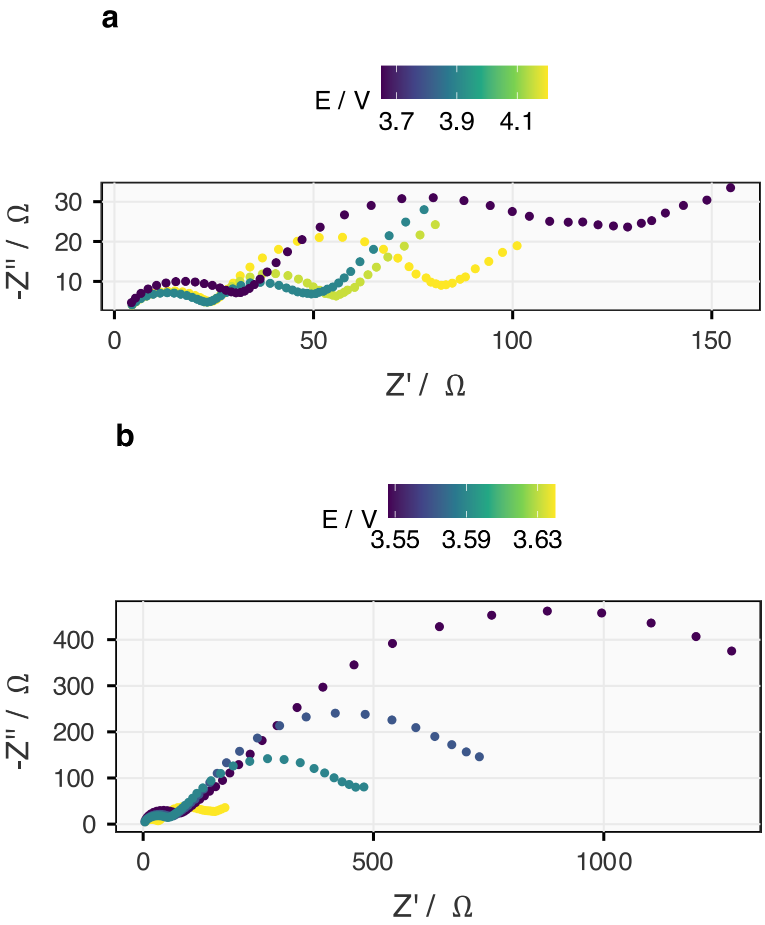

Supplementary Figure 10. Selected impedance spectra from the first discharge of Cell 1. The impedance spectra measured when the working electrode potential is above and below 3.7 V are presented in Nyquist plots in panels **a** and **b**, respectively. Z' and Z'' are the real and imaginary parts of the impedance.

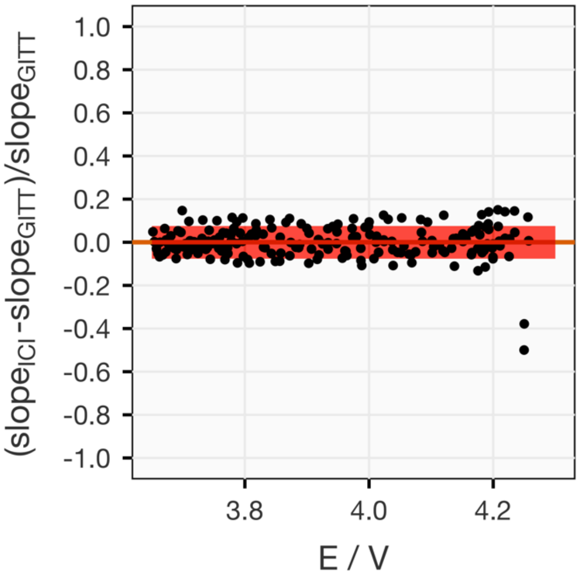

Supplementary Figure 11. Statistical analysis on the relative difference between the slopes of OCP from the GITT and ICI methods.

The relative difference between the slope of the OCP (denoted as dE_{OC}/dt_{I} in Figure 4 in the main text) from the GITT and ICI methods ($slope_{GITT}$ and $slope_{ICI}$, respectively) above 3.65 V in Figure 4 (Cell1 in cycle 1) plotted against the OCP of the electrode (E) against Li/Li^{+}. The average, 0.00021, is shown by the horizontal line and the standard deviation, 0.076, is shown by the height of the shaded area above and below the average value.

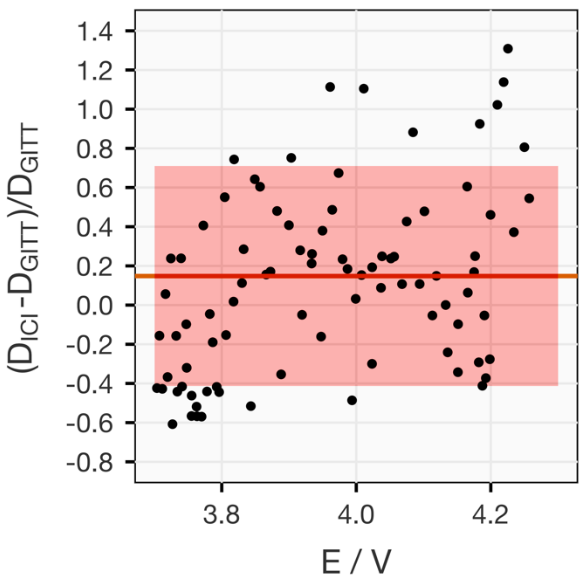

Supplementary Figure 12. Statistical analysis on the relative difference between the diffusion coefficients from the GITT and ICI methods.

The relative difference between the Li-ion diffusion coefficient in NMC811 from the GITT and ICI methods (D_{GITT} and D_{ICI}, respectively) above 3.7 V in Figure 5 (Cell1 in cycle 1) plotted against the OCP of the electrode (E) against Li/Li^{+}. The average, 0.15, is shown by the horizontal line and the standard deviation, 0.56, is shown by the height of the shaded area above and below the average value.

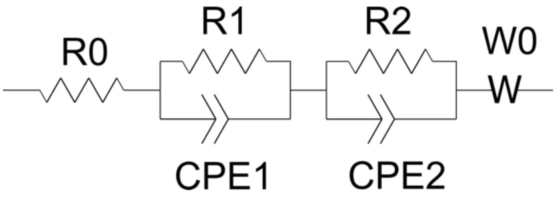

Supplementary Figure 13. Equivalent circuit model used to fit the impedance spectra above 3.7 V. Details of the interpretation of the model are stated in the source of the model.[1]

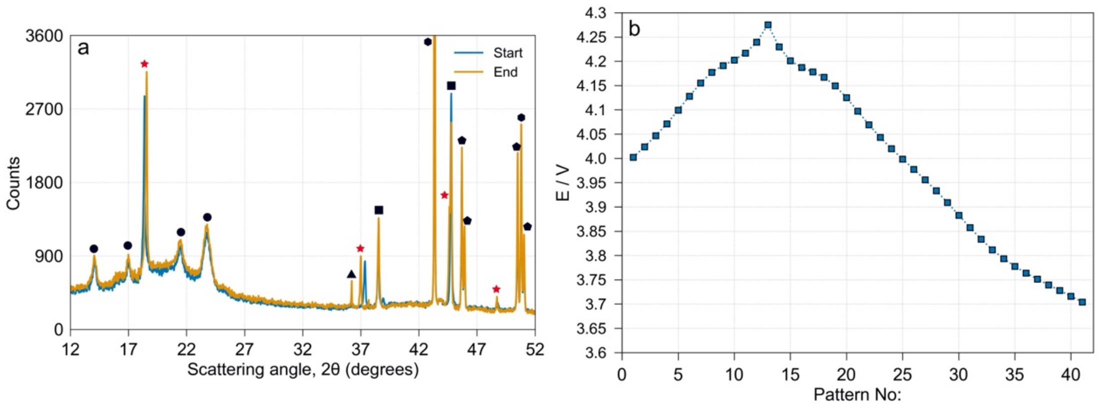

Supplementary Figure 14. Information on the experimental setup of the operando X-ray diffraction (XRD).
a XRD patterns at the beginning (pattern number 1 in **b**) and end (pattern number 41 in **b**) of the operando run. Peaks from NMC811 (star), pouch cell/separators (circles), Li metal (triangles), Al from the tabs and the pouch (squares), Be discs (pentagons) and Cu tabs (hexagon) are highlighted. **b** The electrode potential (E) against Li/Li^{+} is plotted versus the XRD pattern number (Pattern No.), showing the potential measured at the midpoint of the duration of each XRD measurement.

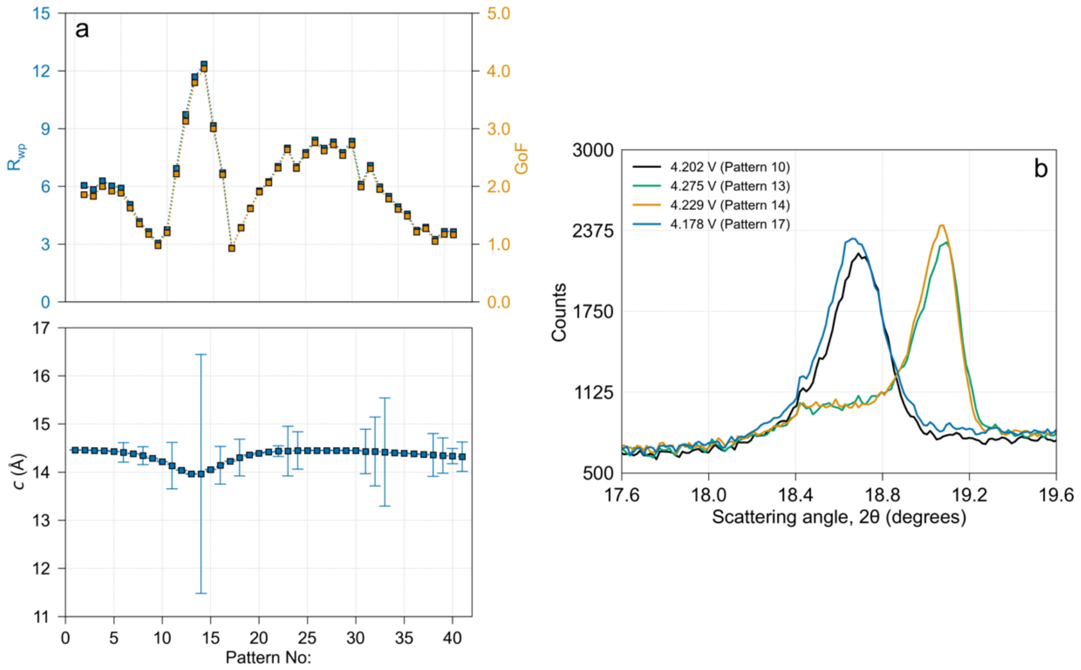

Supplementary Figure 15. Results from the sequential refinements with a single R$\overline{3}$m NMC811 phase and selected XRD patterns near the end of charging and the beginning of discharging.

a R-weighted pattern (R_{wp}) and goodness of fit (GoF) values (top) and *c* lattice parameters (bottom) from the sequential refinements. Note that, in certain cases, the estimated standard deviation is large due to the poor fit of the model to the data. **b** Two patterns at the end of charge (pattern 13 and 14) are compared to ones before (pattern 10) and after (pattern 17). It is clearly seen that patterns 13 and 14 are composed of more than one phase.

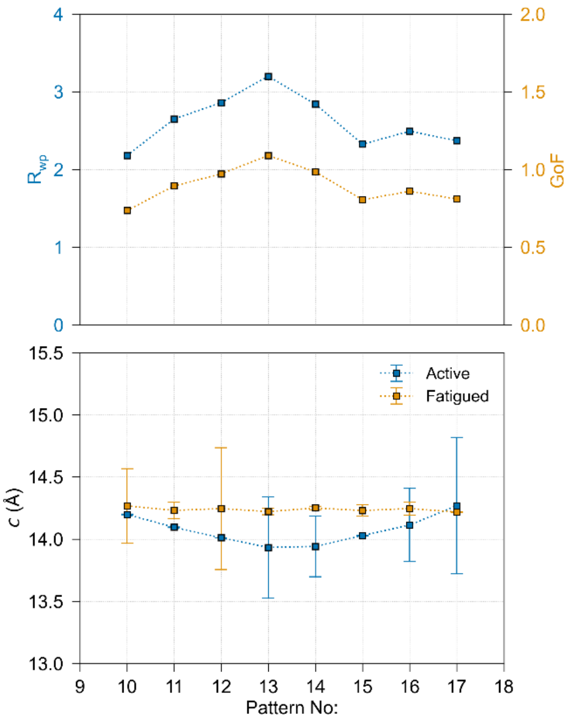

Supplementary Figure 16. Results from the sequential refinements with two R$\bar{3}$m phases as reported by Xu et al.[2]
R-weighted pattern (R_{wp}) and goodness of fit (GoF) values (top) and *c* lattice parameters of the active and fatigued R$\bar{3}$m phases (bottom) from the sequential refinements.

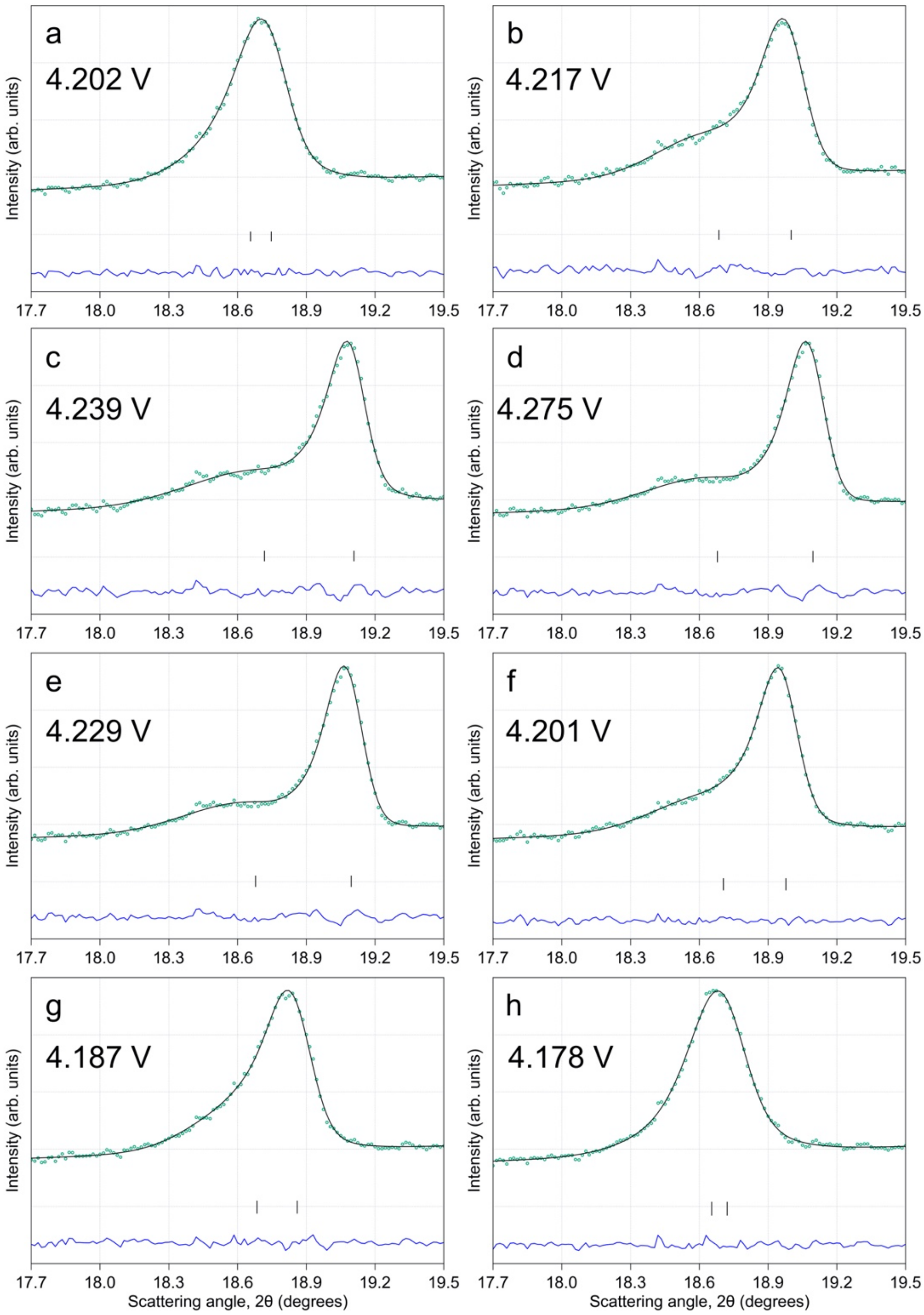

Supplementary Figure 17. Rietveld refinement plots at the end of charging and the beginning of discharging.
Panels **a** to **h** correspond to XRD patterns with pattern numbers 10 to 17, of which the refinement results with *two* $R\bar{3}m$ phases are shown in Supplementary Fig. 16. The measured values, fitted values, residuals and the peak positions of the model are plotted in green dots, black line, blue line and gray rods, respectively. The electrode potential measured at the midpoint of the collection time of each pattern is shown at the top-left corner.

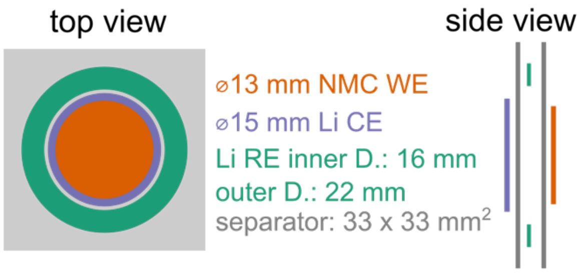

Supplementary Figure 18. Top and side views of schematic illustration of the cell setup. WE, CE and RE denote working, counter and reference electrodes, respectively.

Supplementary Note 1: Full solution to Fick's Second Law and the limit of the current interruption time (Δt) of an ICI measurement

Equations 3, 4 and 5 in the main text can be rewritten as the following, with $n = 1$ for Li-ions:

$$\frac{\partial C(r,t)}{\partial t} = \frac{1}{r^2} D \frac{\partial}{\partial r} r^2 \frac{\partial C(r,t)}{\partial r} \qquad 1$$

$$\begin{cases} -D\dfrac{\partial C}{\partial r} = \dfrac{I}{FA} \\ C(r,0) = C_0 \end{cases} \qquad 2$$

The above has been solved previously[3–6] and is summarized[7,8] as follows for the surface concentration of an electrode particle with a radius r_p:

$$c(r_p, t) = C_0 - \frac{I r_p}{FAD} f\left(\frac{Dt}{r_p^2}\right) \qquad 3$$

where f is defined as follows:

$$f(x) = 3x + 0.2 - 2\sum_{m=1}^{\infty} \frac{1}{\alpha_m^2} e^{-\alpha_m^2 x} \qquad 4$$

where, α_m are the positive roots of $\alpha = \tan(\alpha)$, which is listed elsewhere.[5] $f(x)$ has asymptotes in both positive and negative directions. Within 5% error, Equation 3 can be approximated by the following:

$$f(x) = \begin{cases} \dfrac{2}{\sqrt{\pi}}\sqrt{x}, & x < 0.0032 \\ 3x + 0.2, & x > 1.27 \end{cases} \qquad 5$$

It can be observed that when $x < 0.0032$, Equation 3 can be reduced to

$$C(r_p, t) = C_0 - \frac{2I\sqrt{t}}{FA\sqrt{D\pi}} \qquad 6$$

which is the same as Equation 5 in the main text.
The ICI method is derived on the assumption that $F(\tau_1+\Delta t) \approx F(\tau_1)$ in Equations 14 and 15 in the main text. Suppose a relative error of δ is allowed, the two terms should satisfy the following;

$$1 - \frac{F(\tau_1)}{F(\tau_1 + \Delta t)} < \delta \ or \ \frac{F(\tau_1)}{F(\tau_1 + \Delta t)} > 1 - \delta \qquad 7$$

From Equation 3 in this section, it is known that

$$F(t) = -\frac{I r_p}{FAD} f\left(\frac{Dt}{r_p^2}\right) \qquad 8$$

where, the function and parameters are explained previously. The error can be discussed in terms of both asymptotes of f in Equation 5 since the values are bounded by the asymptotes, which can be found out by plotting the function. Thus, for $\tau_1 < 0.0032 r_p^2/D$,

$$F(t) = -\frac{2I}{FA\sqrt{\pi D}}\sqrt{t} \qquad 9$$

$$1 - \delta < \frac{\sqrt{\tau_1}}{\sqrt{\tau_1 + \Delta t}} \qquad 10$$

$$\rightarrow \Delta t < \frac{(2\delta - \delta^2)}{(1-\delta)^2}\tau_1 \qquad 11$$

Let, $\delta = 0.03$, $\tau_1 = 600$ s. The maximum Δt is around 37 s.
For $\tau_1 > 1.27 r_p^2/D$,

$$F(t) = -\frac{I}{FA}\left(\frac{3t}{r_p} + \frac{r_p}{5D}\right) \qquad 12$$

$$(1-\delta) < \frac{\dfrac{3\tau_1}{r_p} + \dfrac{r_p}{5D}}{\dfrac{3\tau_1 + 3\Delta t}{r_p} + \dfrac{r_p}{5D}} \qquad 13$$

$$\rightarrow \Delta t < \frac{\delta}{1-\delta}\left(\tau_1 + \frac{r_p^2}{15D}\right) \qquad 14$$

With $\delta = 0.03$, $\tau_1 = 600$ s, $r_p = 2\ \mu$m and $D = 10^{-11}$ cm^2 s^{-1}. The maximum Δt is around 26 s.
Since the $\tau_1 > 1.27 r_p^2/D$ is more likely and Equation 12 is stricter than Equation 10, considering only Equation 12 is sufficient in most cases. Nonetheless, in either case, the $\Delta t = 5$ s in the ICI analysis of this work should be considerably below the limit.

It is worth noting that in the standard ICI protocol, the current is only stopped for a few seconds at a time, in contrast to GITT. Therefore, the limit of Δt increases over the number of measurements in the same course of charge or discharge since, τ_1 can be effectively accumulated due to the transient current pauses. The limit of Δt discussed above is thus a conservative estimation for the standard ICI protocol.

Supplementary Note 2: Determination of data collection intervals for $dE/d\sqrt{t}$

With Equation 5 in Supplementary Note 1, the impact of the choice of data selection interval for the GITT analyses can be examined. With $r = 2$ μm (average particle radius) and $D = 10^{-11}$ cm^2 s^{-1} (which is the maximum value in Figure 5 in the main text), the maximum t is 12.8 s. However, if only potential measurements within 12.8 s are selected, the linear regression renders very large standard deviations due to the limited number of data points. Therefore, the upper limit of the interval is set at 40 s after confirming that the E-$\sqrt{t}$ plots remain linear. The lower limit is set at 5 s since there is a transition region before the linear region on some of the E-$\sqrt{t}$ plots, as reported in previous GITT works on $LiNi_{0.5}Mn_{0.3}Co_{0.2}O_2$ (NMC523).[7] This transition region is prolonged below 3.7 V, as shown in Supplementary Fig. 8a. The phenomenon is also observed in EIS measurements in this work and in the literature for $LiNi_{0.33}Mn_{0.33}Co_{0.33}O_2$ (NMC111),[1,9] where the Warburg element shifts to lower frequencies. Thus, as the cell is discharged below 3.7 V, the linear region on the E-$\sqrt{t}$ plots shifts to 50–150 s. The semi-infinite diffusion assumption still holds here because D is significantly lower here. The situation is more complicated when the cell is charged in the same SoC because the transition region is still long, but D derived from 50–150 s is around $5 \cdot 10^{-11}$ cm^2 s^{-1}, which makes 150 s considerably long for the semi-infinite diffusion assumption. Therefore, fitting the data to the full solution in Equations 3 and 4 in Supplementary Note 1 is necessary to derive the diffusion coefficient at SoC below 3.7 V upon charging.

Supplementary Note 3. Analysis of the electrochemical impedance spectra

The impedance spectra above 3.7 V were fitted to the equivalent circuit model (ECM) in Supplementary Fig. 13. The ECM is adopted from a previous work on NMC111[1] but the finite-space Warburg element is substituted here by a semi-infinite Warburg element because the spectra with the lowest frequency at 10 mHz do not show a vertical tail. The spectra below 3.7 V show enlarged second semicircle (R2 and CPE2) and do not possess sufficient data points to fit the Warburg element, as shown in Supplementary Fig. 10. Some spectra between 3.6 and 3.7 V from Cell 2 can be fitted to the ECM but the majority of the spectra from Cell 2 show a depressed Warburg element with an phase angle of about 22.5°. Therefore, the resulting *k* and *R* from the EIS fittings of Cell 2 should be interpreted with caution. Complete set of impedance spectra can be found via Zenodo.[10]

Supplementary Note 4: Details of the analysis of operando X-ray diffraction (XRD) data

The experimental details of the operando XRD measurement on Cell 1 are specified in the Method section in the main text. Diffraction patterns at the start and end of the operando run, with all the peaks ascribed to the corresponding cell components, are shown in Supplementary Fig. 14a. The peaks arise from the pouch cell/separators (polyethylene and polypropylene, circles), Li metal (triangles), Al from the tabs and the pouch (square), Be discs (pentagon) and Cu tabs (hexagon). The peaks from these components do not undergo any noticeable change upon cycling. Four peaks from the rhombohedral ($R\bar{3}m$) NMC811 material (stars) are also visible. However, only the 003 reflection (~18° 2θ) is distinct and free from overlap with peaks from other cell components. Therefore, Rietveld refinements[11,12] of the NMC811 structure were carried out against the 003 reflection. As the objective of the refinements were to track the changes in the *c* lattice parameter of the $R\bar{3}m$ phase and to distinguish the "active" and the "fatigued" phases,[2] structural refinements using only the 003 reflection is sufficient. As a guide to interpreting the results from the refinements, the evolution of voltage (E) as a function of the XRD pattern no: is shown in Supplementary Fig. 14b.

Rietveld refinements were carried out using the Topas-Academic (V6) software.[13] A second-degree Chebychev polynomial function was used to fit the background. The profile of the 003 reflection was modeled using a Thompson-Cox-Hastings pseudo-Voigt function (*TCHZ_Peak_Type*) together with a peak asymmetry function (*Simple_Axial_Model*), to account for the axial divergence imparted by the diffractometer setup. The latter was calibrated using a NIST Si 640c standard reference material and was fixed during the refinements. The NMC811 structure model used was similar to the one used in a previous study.[14] The peak shape functions and the refinement metrics mentioned in the following sections are defined in the Topas-Academic manual.

Supplementary Note 5: Discussion about single and dual $R\bar{3}m$ phase sequential refinements

Results from the sequential Rietveld refinements of a single $R\bar{3}m$ NMC811 phase are shown in Supplementary Fig. 15a. In the top panel, the R-weighted pattern (R_{wp}) and goodness of fit (GoF) values are shown, where it is seen that these metrics increase substantially between pattern numbers 10–17. The bottom panel shows the evolution of *c* lattice parameter. Here, the expected trend of shrinking *c* at high SoC and the recovery upon discharging is seen. Note that, in certain cases, the estimated standard deviation is large due to the poor fit of the model to the data. This is further evidenced in Supplementary Fig. 15b where two patterns at the end of charge (pattern 13 and 14) are compared to ones before (pattern 10) and after (pattern 17). It is clearly seen that patterns 13 and 14 are composed of more than one phase.
To confirm the presence of the “active” and “fatigued” $R\bar{3}m$ phases as reported by Xu et al.,[2] Rietveld refinement of two phases against the 003 reflection from patterns 10–17 were carried out. In Supplementary Fig. 16, it can be seen that an improved fit is obtained with dual $R\bar{3}m$ NMC811 phases.

Supplementary References

1. Charbonneau, V., Lasia, A. & Brisard, G. Impedance studies of Li^+ diffusion in nickel manganese cobalt oxide (NMC) during charge/discharge cycles. *Journal of Electroanalytical Chemistry* **875**, 113944 (2020).
2. Xu, C. *et al.* Bulk fatigue induced by surface reconstruction in layered Ni-rich cathodes for Li-ion batteries. *Nat Mater* **20**, 84–92 (2021).
3. Subramanian, V. R., Ritter, J. A. & White, R. E. Approximate Solutions for Galvanostatic Discharge of Spherical Particles I. Constant Diffusion Coefficient. *J Electrochem Soc* **148**, E444 (2001).
4. Subramanian, V. R. & White, R. E. New separation of variables method for composite electrodes with galvanostatic boundary conditions. *J Power Sources* **96**, 385–395 (2001).
5. Liu, S. An analytical solution to Li/Li+ insertion into a porous electrode. *Solid State Ion* **177**, 53–58 (2006).
6. Carslaw, H. S. & Jaeger, J. C. *Conduction of heat in solids*. (Clarendon, 1986).
7. Nickol, A. *et al.* GITT Analysis of Lithium Insertion Cathodes for Determining the Lithium Diffusion Coefficient at Low Temperature: Challenges and Pitfalls. *J Electrochem Soc* **167**, 090546 (2020).
8. Delacourt, C., Ati, M. & Tarascon, J. M. Measurement of Lithium Diffusion Coefficient in LiyFeSO4F. *J Electrochem Soc* **158**, A741 (2011).
9. Shaju, K. M., Subba Rao, G. V. & Chowdari, B. V. R. Influence of Li-Ion Kinetics in the Cathodic Performance of Layered $Li(Ni_{1/3}Co_{1/3}Mn_{1/3})O_2$. *J Electrochem Soc* **151**, A1324 (2004).
10. Chien, Y.-C. *et al.* Supporting data for 'Rapid determination of solid-state diffusion coefficients in Li-based batteries via intermittent current interruption method'. Zenodo. https://doi.org/10.5281/zenodo.4964673 (2021).
11. Rietveld, H. M. Line profiles of neutron powder-diffraction peaks for structure refinement. *Acta Crystallogr* **22**, 151–152 (1967).
12. Rietveld, H. M. A profile refinement method for nuclear and magnetic structures. *J Appl Crystallogr* **2**, 65–71 (1969).
13. Coelho, A. A. TOPAS and TOPAS-Academic: An optimization program integrating computer algebra and crystallographic objects written in C++: An. *J Appl Crystallogr* **51**, 210–218 (2018).
14. Liu, H. *et al.* Understanding the Roles of Tris(trimethylsilyl) Phosphite (TMSPi) in $LiNi_{0.8}Mn_{0.1}Co_{0.1}O_2$ (NMC811)/Silicon–Graphite (Si–Gr) Lithium-Ion Batteries. *Adv Mater Interfaces* **7**, 2000277 (2020).
